# Galectin-3 shapes microglial phenotype through endogenous and exogenous mechanisms

**DOI:** 10.3389/fncel.2025.1729776

**Published:** 2025-12-18

**Authors:** Lluís Camprubí-Ferrer, Yiyi Yang, Rosalía Fernández-Calle, Antonio Boza-Serrano, Juan García-Revilla, Javier Frontiñán-Rubio, Tomas Deierborg

**Affiliations:** 1 Experimental Neuroinflammation Laboratory, Department of Experimental Medical Sciences, Lund University, Lund, Sweden; 2 Instituto de Biomedicina de Sevilla, IBiS/Hospital Universitario Virgen del Rocío/CSIC/Universidad de Sevilla, Seville, Spain; 3 Departamento de Bioquímica y Biología Molecular, Facultad de Farmacia, Universidad de Sevilla, Seville, Spain; 4 Oxidative Stress and Neurodegeneration Group, Faculty of Medicine, Universidad de Castilla-La Mancha, Ciudad Real, Spain

**Keywords:** CLEC7A, endocytosis, galectin-3, microglia, neuroinflammation, phagocytosis

## Abstract

Galectin-3 (Gal3) is a multifunctional lectin expressed and released by microglia, where it influences diverse processes in both homeostasis and disease. To dissect its intracellular and extracellular roles, we generated Gal3-deficient BV2 microglial cells and systematically assessed how genetic deletion and exogenously added recombinant Gal3 shape microglial physiology. Gal3 deletion increased cell area, mitochondrial activity, and motility without affecting proliferation, linking endogenous Gal3 to microglial energetic control and dynamic cellular physiology. Endogenous Gal3 was required to maintain CD11b surface levels, and restrains TREM2 and Clec7a expression, whereas exogenous Gal3 promoted CD45 internalization and drove a paracrine TNFα release. Endogenous and exogenous Gal3 are synergistically needed for Syk phosphorylation and NOX2 expression. Internalization assays demonstrated that endogenous Gal3 constrained phagocytosis and endocytosis, while exogenous Gal3 enhanced endocytosis in a paracrine manner. In the Alzheimer’s disease 5xFAD mouse model, where Gal3 deletion was reported to lower amyloid plaque burden, the absence of Gal3 does not affect microgliosis but elevates Clec7a levels around plaques. Together, these findings reveal Gal3 as a critical regulator of microglial homeostasis, uptake pathways, receptor expression, and inflammatory signaling. We have defined a novel microglial regulation based on endogenous and exogenous pools of Gal3. By identifying a novel Gal3-Clec7a interaction, this work highlights Gal3 as a key modulator of microglial phenotype and a potential target for therapeutic modulation of neuroinflammation.

## Introduction

Alzheimer’s disease (AD) is the most common neurodegenerative disorder, marked by the progressive accumulation of amyloid-β (Aβ) plaques, neurofibrillary tangles composed of hyperphosphorylated tau, and chronic neuroinflammation (Serrano-Pozo et al., 2011; Hampel et al., 2021). Central to AD pathogenesis is the dynamic role of microglia, the brain’s resident immune cells, which undergo profound phenotypic and functional changes during disease progression (Leng and Edison, 2021; Hansen et al., 2018; Heneka et al., 2025). While microglia initially exert neuroprotective effects through Aβ clearance and tissue homeostasis, their sustained activation promotes chronic inflammation, synaptic loss, and neuronal death (Millet et al., 2024; Baligacs et al., 2024; Clayton et al., 2021; Valiukas et al., 2025; Bachiller et al., 2018).

Recent advances in single-cell transcriptomics have identified distinct microglial subpopulations in neurodegenerative conditions in mice, most notably disease-associated microglia (DAM) (Valiukas et al., 2025; Krasemann et al., 2017; Keren-Shaul et al., 2017). These phenotypes arise in response to pathological stimuli and are characterized by altered gene expression profiles. A common feature across these activated states is the upregulation of galectin-3 (Gal3), a β-galactoside-binding lectin that has emerged as a critical regulator of microglial activation and function in neurodegenerative diseases (Garcia-Revilla et al., 2022; Lozinski et al., 2024). As we previously have reported, Gal3 is primarily expressed and released by reactive microglia and has been implicated in multiple aspects of AD pathology, ranging from amyloid aggregation to neuroinflammatory signaling, along with increased CSF levels in AD patients (Burguillos et al., 2015; Boza-Serrano et al., 2019; Tao et al., 2020). Based on this data monoclonal antibody targeting Gal3 is being tested for AD in clinical phase 1b/2a (TrueBinding Inc.; ClinicalTrials.gov identifiers: NCT05074498 and NCT05476783) with no published phase 2 results yet.

Gal3 regulates key microglial processes including phagocytosis, endocytosis, morphology, and cytokine release, acting both intracellularly and extracellularly (Rotshenker, 2022; Garcia-Revilla et al., 2024; Boucheham et al., 2025; Tabel et al., 2022; Prins et al., 2024). The clinical importance of Gal3 in AD is underscored by genetic and pathological evidence: LGALS3 variants are linked to increased AD risk, Gal3 is elevated in patient brains, including genetic and early onset AD (Rotshenker, 2022), and enriched in plaque-associated microglia, and functionally, it promotes amyloid aggregation and potentiates TREM2-DAP12 signaling (Boza-Serrano et al., 2019). Together, these findings position Gal3 as a central mediator linking microglial activation to AD pathology.

Gal3’s interactions with key AD-associated receptors further underscore its importance. TREM2, a receptor with established genetic ties to AD, binds Gal3 directly, a partnership critical for microglial survival, metabolic regulation, and the emergence of DAM phenotypes around plaques (Boza-Serrano et al., 2019). Gal3 also modulates TLR4-mediated inflammatory cascades, which are central to Aβ-induced microglial activation and chronic neuroinflammation (Burguillos et al., 2015; Walter et al., 2007; Papageorgiou et al., 2016). Moreover, the C-type lectin receptor Clec7a (Dectin-1), another receptor implicated in AD, binds Aβ and mediates pro-inflammatory responses via Syk/NF-κB signaling (Zhao et al., 2023), a pathway reported to be influenced by Gal3 in other cell types (Chen et al., 2022; Esteban et al., 2011).

In this study, we investigate the multifaceted role of Gal3 in microglial biology within the context of AD. Knowing Gal3’s importance in cellular functions endogenously as well as being released from microglia, we focused on the consequences of deleting endogenous Gal3 as well as pre-treating with exogenous recombinant Gal3 protein. We studied its regulation of phagocytic and endocytic pathways, its impact on inflammatory responses, and its interactions with disease-associated receptors, such as Clec7a. By dissecting these mechanisms, we aim to understand how Gal3 shapes microglial function and to identify strategies that could harness protective microglial responses while limiting the harmful consequences of chronic neuroinflammation in AD.

## Materials and methods

### Galectin-3 protein production

Recombinant human galectin-3 (Gal3) was produced by the Lund Protein Production Platform (LP3). Gal3 production were prepared as previously described (Salomonsson et al., 2010). Briefly, Gal3 production was performed in strain *E. coli* TUNER(DE3)/pET3c-hum-Gal3 grown in LB medium, 18 °C, 250 rpm with 1 mM IPTG overnight. After cell lysis and ultracentrifugation, Gal3 was purified on a 20-ml lactocyl-sepharose column. Peak fractions containing Gal3 were pooled and dialyzed against phosphate buffer saline (PBS, Nzytech), pH 7.4 (Garcia-Revilla et al., 2023). Freeze-dried Gal3 was stored at −80 °C until use. Supplementary Figure 1a shows SDS-PAGE analysis of the final Gal3 product.

### Cell lines

Murine microglial BV2 cell lines (RRID: CVCL_0182) were cultured in Dulbecco’s Modified Eagle Medium GlutaMax (DMEM GlutaMax, Gibco, Cat: 61965-059) supplemented with 10% heat inactivated Fetal Bovine Serum (FBS, Gibco, Cat: 17593595) and 1% penicillin–streptomycin (Cytiva, Cat: SV30010) and kept at 37 °C 5% CO_2_. Galectin-3 knockout BV2 (Gal3KO) cells were generated with the Alt-R CRISPR-Cas9 System (Integrated DNA Technologies, IDT), following manufacturer’s instructions. Briefly, two Alt-R CRISPR-Cas9 CRISPR RNA (crRNA, IDT) guides were designed to excise the transcription starting site of the galectin-3 gene (guide 1: CTTCTAGTACTCTTTTCCCC, guide 2: TACCCGCAGGAGAGCCAAGG, IDT; protospacer-adjacent motif sequences not included). Each one was then mixed with trans-activating Alt-R® CRISPR-Cas9 CRISPR RNA (tracRNA, IDT) to form the single-guide RNA (sgRNA). Afterward, ribonucleoprotein (RNP) complex was formed by mixing Alt-R S.p. HiFi Cas9 Nuclease V3 (IDT) with both sgRNAs in Opti-MEM (Gibco, Cat: 31985070). Reverse transfection was performed by mixing the RNP complex with Lipofectamine RNAiMAX (Invitrogen) and added to a 96-well plate, where 40.000 BV2 cells were then seeded. The same procedure was carried out as negative control with the RNP complex without the crRNA (WT cells). After that, propidium iodide (PI)-negative single-cells (1:1000 dilution, BioLegend, Cat: 421301, 5 min incubation) were sorted using the BD FACSAria™ III Cell Sorter (BD Biosciences) in new 96-well plates containing one third of conditioned medium to promote cell growth to create separate colonies. When wells were >95% confluent, individual clones were seeded in 24-well plates to perform downstream experiments to assess the success of the knockout. Clone 3 was used for all subsequent experiments.

### Live-cell imaging

For live-cell imaging experiments, 7,000 cells were seeded on a 96-well PhenoPlate microplate (Revvity, Cat: 6055302). After cellular attachment to the plate bottom, 1 μM of Gal3 protein or PBS was added 24 h prior to initiating imaging (preGal3). The following intracellular compartments were labeled through the addition of cell-penetrating dyes: mitochondria (using MitoTracker Deep Red FM, Invitrogen, Cat: M22426, 1:2500 dilution, 30 min incubation), lysosomes (using LysoTracker Green DND-26, Invitrogen, 1:2500 dilution, 5 min incubation) and nuclei (using Hoechst 33342, Invitrogen, Cat: H3570, 1:1000 dilution, 10 min). Eight images per well were taken every 30 min for 20 h with Operetta CLS High Content Screening (Revvity) by confocal spinning disk technology with a 40 × water immersion objective, and cells were kept at 37 °C, 5% CO_2_ for the duration of all experiments. Superoxide production analysis was performed by incubation of the cells with MitoSox 5 μM (MitoSox Red, Invitrogen, Cat: M36007) under the same live-cell imaging parameters, after, the images were acquired 30 min after incubation.

### Motility assay

2,500 cells were seeded on 96-well PhenoPlate microplates (Revvity, Cat: 6055302). Pretreatment for 24 h with Gal3 was performed as described. Nuclei were stained with Hoechst 33342 for 5 min, and live-cell imaging was conducted by brightfield and Hoechst (at 350 nm excitation) channels at 15-min intervals over a 6-h period using Operetta CLS System (Revvity). Cells were kept at 37 °C, 5% CO_2_ for the duration of the experiment. Displacement and total accumulated distance were analyzed using Operetta CLS Harmony Software (at least 250 cells per replicate).

### Flow cytometry

For flow cytometry experiments, cells were detached by removing medium, washing once with sterile PBS and adding trypsin 2.5% (Gibco, Cat: 15090046) for 2 min. Cells were then spun down in fresh medium and resuspended in FACS buffer (PBS + 2% FBS). A total of 20,000 events per sample of the target population were analyzed in BD FACSAria III Cell Sorter. Data was analyzed with FlowJo software version 10.4.0.

For cell division assay, 100,000 cells were seeded on 24-well plates. They were stained with CFSE (CFSE Cell Division Assay Kit, Cayman Chemical, Cat: 154-10009853) according to the manufacturer’s instructions. 4 h after the labeling, cells were collected from 24-well plates and stained for 5 min with PI (BioLegend, Cat: 421301, 1:1000 dilution). The live (PI-negative population) cells were then gated for proliferation assessment with FACSAria III cytometer. The cells that divided were determined as a percentage of CFSE-positive cells below the intensity peak of cells with CFSE at 0 h.

For CD11b and CD45 receptors characterization in WT and Gal3KO cells with preGal3, 50,000 cells were seeded on 48-well plates and pretreated with preGal3 for 24 h, Fc-receptors were blocked using TruStain FcX™ (anti-mouse CD16/CD32) antibody (1:500, BioLegend, Cat: 101319) for 5 min on ice. Afterward, they were stained with rat anti-mouse CD45-AlexaFluor 488 (1:200 dilution, BioLegend, Cat: 110717) and rat anti-mouse CD11b-PE/CY7 (1:200 dilution, Biosite, Cat: ASB-XQYMH4-0.1). PI (BioLegend, Cat: 421301, 1:1000 dilution, 5 min) was used as viability dye for cells. Supplementary Figure 3a shows the gating strategy followed to discard doublets and dead cells to reach the target population in all flow cytometry experiments, utilizing CD45 and CD11b staining as example. Supplementary Figure 3b shows median fluorescence intensity (MFI) histograms of CD11b and CD45 stainings.

### Immunoassay plate

V-PLEX Proinflammatory Panel 1 (mouse) Kit (Cat: K15048D) or U-PLEX Metabolic Group 1 (mouse) Assay (Cat: K152ACM) from MesoScale Discovery (MSD) were used to evaluate cytokine levels in media from cell cultures. V-PLEX plate included IFN-*γ*, IL-1β, IL-2, IL-4, IL-5, IL-6, KC/GRO, IL-10, IL-12p70 and TNFα; U-PLEX plate included IFN-γ, IL-1β, IL-2, IL-4, IL-10, IL-12p70, IL-13, TNFα and MMP-9. Assays were performed following manufacturer’s instructions and read using the MSD 1300 Meso Quickplex SQ 120MM Imager with Methodical Mind software, and analyzed with Discovery Workbench software (MSD). TNFα was the only cytokine detected within the detection range consistently throughout all samples both in the V-PLEX and U-PLEX panels.

### Phagocytosis and endocytosis assays

7,000 cells were seeded in a 96-well PhenoPlate microplates (Revvity, Cat: 6055302) to test their phagocytic and endocytic ability. To study phagocytosis, Zymosan A (*Saccharomyces cerevisiae*) Alexa Fluor 488 conjugated BioParticles (Invitrogen, Cat: Z23373) were then added to the cells for 6 h (1:5000 dilution). To study endocytosis, dextran Alexa Fluor 647 particles (Invitrogen, Cat: D22914) were added for 20 min (1:4000 dilution). The following intracellular compartments were labeled through the addition of cell-penetrating dyes: mitochondria (using MitoTracker Deep Red FM or MitoTracker Green FM, Invitrogen, Cat: M22426 and Cat: M7514, at 1:2500 dilution), lysosomes (using LysoTracker Green DND-26, Invitrogen, Cat: L7526, at 1:2500 dilution) and nuclei (using Hoechst 33342, Invitrogen, Cat: H3570 at 1:1000 dilution). 1 μM of Gal3 was added either 24 h prior to the addition of zymosan and dextran (preGal3), or coincubated with zymosan for 6 h (coincubation Gal3 + zymosan), or 6 h before dextran addition (preGal3 6 h + dextran). Finally, cells treated with dextran were fixed with 4% paraformaldehyde (PFA, Histolab). For Supplementary Figure 2, imaging was performed in Zeiss 780 confocal laser-scanning microscope (Zeiss) and analysis in Fiji by ImageJ 1.53 software (NIH). Imaging and quantification was performed in Operetta CLS system (Revvity).

### RNA extraction and RT-qPCR from *in vitro* cultures

Total RNA from WT and Gal3KO BV2 cells was extracted with TRI Reagent (Sigma-Aldrich, Cat: AM9738) according to manufacturer’s instructions. RNA was quantified using the Thermo Fisher Nanodrop 2000c spectrophotometer using an extinction coefficient of 0.025 (μg/ml)-1 cm-1 at 260 nm. RNA was reverse-transcribed (500 ng) into cDNA in a total volume of 20 μL with the iScript cDNA synthesis kit (Bio-rad, Cat: 1708890) in the Bio-Rad T100 Thermal Cycler device. qPCRs were performed in 384-well plates in the Bio-Rad CFX384-C1000 Touch Thermal Cycler. 25 ng of cDNA were loaded in the reaction using the SsoAdvanced Universal SYBR Green Supermix (Bio-rad, Cat: 1725270) and the annealing temperature was 60 °C. Primer pairs for the target genes were developed with the Primer BLAST tool from the National Institutes of Health. As reference, gene Hprt or 18S was used. Primers were validated when efficiency was comprised within 80–110%. Relative expression ratio of each target gene was calculated by the ΔΔCt method.

**Table 1:** 

Target gene	Forward sequence 5′ to 3’	Reverse sequence 5′ to 3’
*Hprt*	CTGGTGAAAAGGACCTCTCGAAG	CCAGTTTCACTAATGACACAAACG
*Lgals3 (galectin-3)*	CGGTCAACGATGCTCACCTA	GGCTTAGATCATGGCGTGGT
*18S*	CTCAACACGGGAAACCTCAC	CGCTCCACCAACTAAGAACG
*Tnfa*	TGCCTATGTCTCAGCCTCTTC	GAGGCCATTTGGGAACTTCT
*Il1b*	TGTAATGAAAGACGGCACACC	TCTTCTTTGGGTATTGCTTGG
*Lgals1*	CAATCATGGCCTGTGGTCTGG	TTCCCAGGTTCAGCACAAAGC
*Lgals8*	CCATCGGGTTCAGATTCAGC	ACCTTGCTTCAAATGGCAGG
*Lgals9*	GTCTGCTTGGGAGGATGC	AAGTTCTTCAGGCGGTGG
*Lgals3bp*	GTGGTCTGCTCCAACGATACC	CTGGATGAATAGGTCGCAGCC

### Galectin-1 stimulation

100,000 cells from WT and Gal3KO BV2 were seeded on a 24-well plate (Sarstedt) and treated with 1 μM recombinant Gal1 produced by Lund Protein Production Platform (LP3, Lund University). DMEM FBS-free cell media was collected after 24 h of exposure with Gal1, Gal3 or combination for Immunoasssay plate cytokine determination (see above). Monolayer cells were later lysed for gene expression determination as described above.

### Cell shape analysis after short-term incubation of Gal3

10,000 cells were seeded on Lumox 96-well plate (Sarstedt Cat: 94.6120.096) and treated with Gal3 for either 360, 180, 60, 30 or 10 min and fixed with 4% paraformaldehyde (Histolab) for 20 min at room temperature. To evaluate cell morphology, the cells were stained with ActinRed 555 (Thermo Fisher, Cat: R37112) and Hoechst 33342 reagent. Images were recorded using a Zeiss 780 confocal microscope (×20 magnification).

### Scratch assay

BV2 cells were plated in 24-well plates and cultured to confluence. A cross-shaped scratch was introduced in the cell monolayer using a 200 μL pipette tip, after which the medium was replaced with fresh medium. For the Gal3 pre-treated condition, cells were incubated with Gal3 for 24 h prior to scratching. In the Gal3 post-treated condition, Gal3 was added to the culture medium immediately after the scratch, concomitant with medium replacement. Phase-contrast microscopy images were acquired at 0 h, 1 h, 6 h, and 24 h post-scratch. The percentage of wound closure was quantified using Fiji by ImageJ software, by measuring the open area devoid of migrated cells immediately after scratching and at 24 h (*n* = 3).

### SDS-PAGE Western blot

To study microglial markers, 50,000 cells were seeded in 24-well plate. After 24 h, the medium was changed to starvation medium (medium with 2% FBS) and 1.0 μM of Gal3 (preGal3) was added to the cells. Cells were then treated with RIPA buffer (Fisher Scientific, Cat: 89900) with PhosStop and cOmplete phosphatase and protease inhibitor tablets (Roche, Cat: 4906837001 and 11836170001) on wet ice, extracts were sonicated for 10 s, 80% amplitude, 0.8 cycles and stored at −80 °C. After centrifugation for 10 min at 15,000 g 4 °C, supernatant was stored at −80 °C. For Gal3KO cell characterization, cells were extracted as described above.

Primary antibodies used were: anti-galectin-3 (1:1000 dilution, goat, R&D Systems, Cat: AF1197), anti-TREM-2 (1:750 dilution, sheep, R&D Systems, Cat: AF1729), anti-TLR4 (1:1000 dilution, rabbit, Santa Cruz Biotechnology, Cat: sc-10741), anti-Dectin-1 (1:500 dilution, rabbit, Abcam, Cat: ab140039), anti-β-Actin Peroxidase (1:10000 dilution, mouse, Sigma-Aldrich, Cat: A3854). Secondary antibodies used were: horse anti-goat IgG peroxidase (Vector Labs, Cat: PI-9500-1), rabbit anti-sheep IgG peroxidase (Invitrogen, Cat: 61-8620), goat anti-rabbit IgG peroxidase (Vector Labs, Cat: PI-1000). All secondary antibodies were used at 1:3000. Blot images were taken using ChemiDoc platform (Bio-rad). Stripping was performed utilizing Restore PLUS Western Blot Stripping Buffer (Thermo Fisher). Western Blot band quantification was carried out using Image Lab Software (Bio-Rad).

### ELISA

Cell lysates were normalized at 0.15 mg/mL utilizing Pierce BCA Protein Assay Kit (Thermo Fisher) and analyzed using Mouse Dectin-1/Clec7a ELISA Kit (AssayGenie, Cat: SBRS1334) following manufacturer’s instructions. The detection limit of the kit is determined to be 0.12 ng/mL.

### Cell division phase analysis

Cell cycle phase distribution in BV2 WT and Gal3KO cells was assessed based on nuclear Hoechst intensity using the Operetta CLS high-content imaging system. Cells were classified into three populations (G1, S, and G2/M phases) according to their sum nuclear fluorescence intensity, and the proportion of cells in each phase was quantified. Supplementary Figure 2 presents representative images, the corresponding intensity histogram, and the resulting phase quantifications.

### Animals

5xFAD and 5xFAD/Gal3KO mice used for Clec7a immunostaining and quantification were generated and genotyped according to Boza-Serrano et al. (2019). To generate 5xFAD mice, wild-type dam and hemizygous sire were crossed. Experimental mice were homozygous for Gal3KO and hemizygous for 5xFAD mutation. Animal housing, handling and experiments were performed under international guidelines approved by the Malmö/Lund animal ethics committee (M30-16, Dnr5.8.18-01107/2018). Mice were housed in groups (maximum 5 animals/cage) and maintained in a 12-h light/dark cycle, with free access to food and water. Only female mice were studied.

### Immunofluorescence

#### Cell cultures

BV2 WT and BV2 Gal3KO cells grown in 96-well PhenoPlate microplates were fixed using 4% paraformaldehyde (Histolab) for 15 min, followed by PBS washes. They were then permeabilized using PBS with Tween-20 (T20, Sigma-Aldrich, Cat: P1379, 0.1%) for 10 min, followed by blocking with PBS-T20 (0.1%) and normal donkey serum (NDS, Sigma-Aldrich, Cat: S30, 10%) for 30 min. Cells were then incubated overnight 4 °C in blocking solution. Primary antibodies used: TOM20 (1:500, rabbit, Proteintech, Cat: 11802-1-AP), NDUFB8 (1:250, mouse, Abcam, Cat: ab110242), p47phox (1:100, mouse, Santa Cruz Biotechnology, Cat: sc-17844), p22phox (1:100, rabbit, Abcam, Cat: ab75941), pSyk (1:250, rabbit, Cell Signaling, Cat: 2717S). After primary incubation, cells were washed three times with PBS and incubated with secondary antibody in blocking solution for 1 h at room temperature. Secondary antibodies were donkey-raised Alexa Fluor conjugates (Invitrogen, 1:500 dilution): anti-rabbit Alexa Fluor 488 (Cat: A-21206), anti-mouse Alexa Fluor 568 (Cat: A10037), and anti-rabbit Alexa Fluor 405 (Cat: A48258). After three PBS washes, cells were incubated with Phalloidin-iFluor 647 (Abcam, Cat: ab176759) for 30 min (1X), followed by DAPI staining for 5 min (1:1000, Invitrogen, Cat: 62248) in stainings that Alexa Fluor 405 was not used. Stainings were imaged and quantified using Operetta CLS.

#### Brain sections

For the quantification of Clec7a reacting to plaque pathology, female 5xFAD and 5xFAD Gal3KO mice were deeply anesthetized with 4% isoflurane in oxygen. They were then transcardially perfused with ice-cold PB. Brains were removed and fixed in 4% paraformaldehyde for 24 h, followed by storage in PB 30% sucrose and cryosectioned at 30 μm of thickness and stored in PBS with 30% sucrose with ethylene glycol (Sigma Aldrich, Cat: 102466-1 L). Immunofluorescence staining was performed as follows. Sections were washed with PBS and antigen retrieval with 88% formic acid (Sigma Aldrich, Cat: 33015) was performed for 8 min. After washing with PBS, blocking was performed with 0.25% T20 and 0.3% Triton-X100 (Sigma Aldrich, Cat: T8787) and 10% NDS in PBS for 2 h. After PBS washing, overnight incubation of primary antibodies at 4 °C in blocking solution was performed. Primary antibodies used were: anti-mDectin-1 (1:50, rat, InvivoGen, Cat: mabg-mdect), anti-MOAB2 (1:500, mouse, Biosensis, Cat: M-1586-100), anti-Iba1 (1:500, rabbit, Wako, Cat: 019-19741). The following day, washes with PBS-T20 were performed and appropriate secondary antibodies were incubated in darkness in PBS-T20-NDS for 1 h at room temperature. Secondary antibodies were donkey-raised Alexa Fluor conjugates (Invitrogen, 1:500 dilution): anti-rat Alexa Fluor 488 (Cat: A-21208), anti-mouse Alexa Fluor 568 (Cat: A10037), anti-rabbit Alexa Fluor 647 (Cat: A-31573). After washing with PBS, sections were stained with DAPI solution (1:1000) for 5 min, washed with PBS and then mounted in Super Frost slides (Thermo Fisher) and coverslips (Menzek-Gläser) were added with ProLong Glass Antifade mounting medium (Thermo Fisher, Cat: P36982). A Leica SP8 laser scanning microscope with 63 oil objective was employed to image samples. Multiple images in a z-stack with a 2 μm step size were taken in mouse dentate gyrus. CD45 (1:250, mouse, Novus, Cat: NB500-319) and CD11b (1:250, rat, BioLegend, Cat: 101202) stainings were carried out similarly, except that antigen retrieval and permeabilization steps were omitted, and DAPI was substituted by Methoxy-X04 (0.01 mg/mL, Biotechne, Cat: 4920). Secondary antibodies used were donkey-raised, at 1:500 dilution: Alexa Fluor anti-mouse 488 (Cat: A-21202) and Alexa Fluor anti-rat 568 (Cat: A78946).

### Image analysis

For live-cell and other experiments using Operetta CLS, images were analyzed employing the High Content Analysis System Harmony (Revvity). The results shown are the mean of at least 6 imaged fields per well (with intraexperimental well duplicates). For the rest of image analysis experiments, Fiji by ImageJ software (NIH) was used. Immunofluorescence of mouse brains was analyzed utilizing a background subtraction of 50 pixel per each individual picture in the z-stack, followed by a maximum intensity projection of the channels. For Clec7a and Iba1 quantification, each plaque was manually selected in the MOAB2 channel, and the automatic threshold “Moments” available in Fiji was used quantify the % area covered by Clec7a per plaque. For CD45-CD11b quantification, an 8 μm enlarged area around plaque cores was used to quantify the % area covered by these markers utilizing automatic threshold algorithms (“RenyiEnthropy” was used in CD45 channel and “Triangle” in CD11b channel).

### Statistical analysis

All statistical analyses were performed using GraphPad Prism 10. A significance threshold of *p* < 0.05 was applied. Exact *p*-values, including those approaching significance, are reported in the figure panels or legends. The specific statistical tests used for each dataset are indicated in the corresponding figure legends. For full ANOVA results and detailed test outputs, see Supplementary Table 1.

## Results

### Endogenous galectin-3 is efficiently deleted in BV2 microglial cells

**Figure 1:**
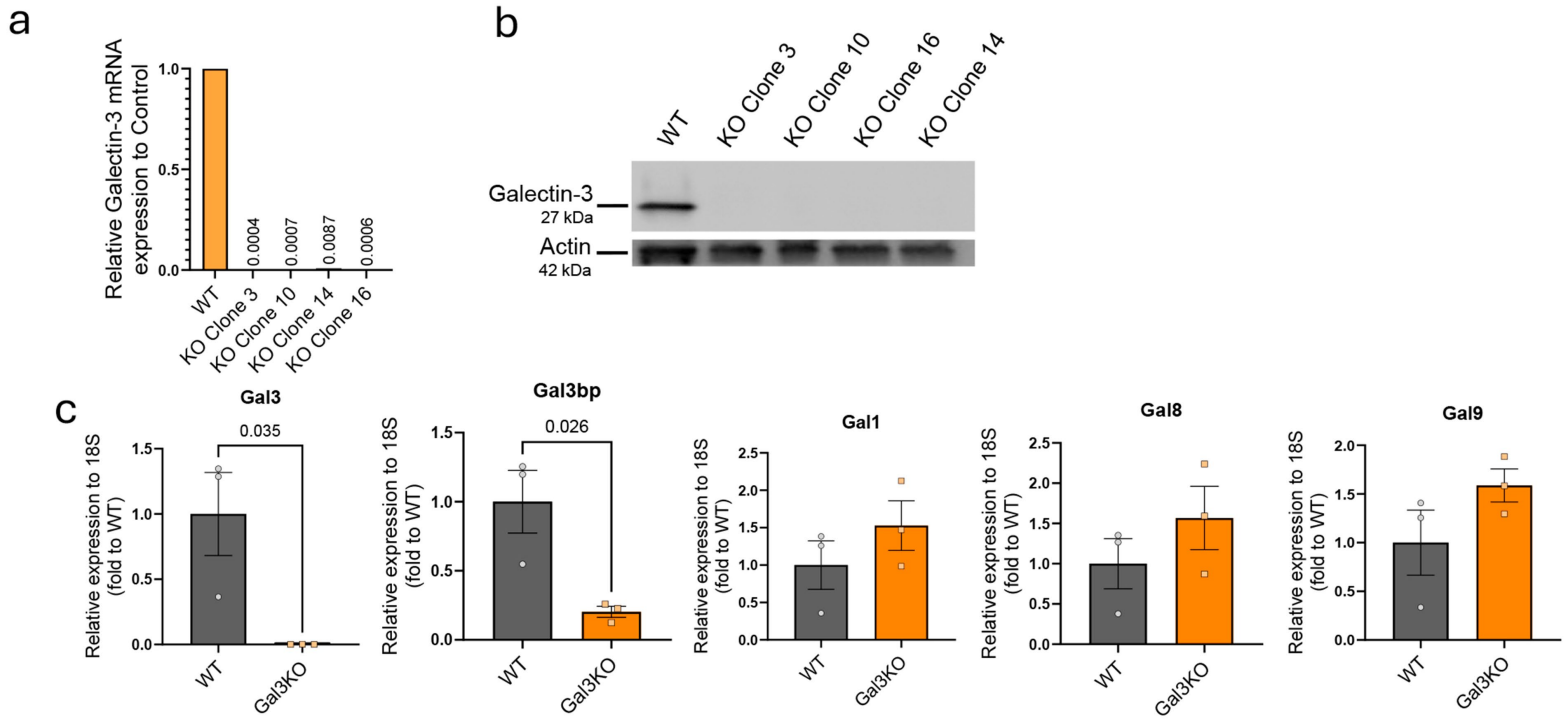
Characterization of BV2 cell line with galectin-3 knockout. **(a)** RT-qPCR analysis of Gal3 mRNA expression in BV2 clones generated with the Alt-R CRISPR-Cas9 system. **(b)** Western blot confirming loss of Gal3 protein in BV2 CRISPR clones. **(c)** RT-qPCR analysis of other galectin family members in Gal3KO cells (clone 3 used for subsequent experiments). Data are shown as individual replicates (each experimental replicate corresponds to an independent culture performed from a different cell passage) with mean ± SEM. In **(c)**, unpaired parametric *t*-test was used. *p*-values are expressed with 3 decimals.

Galectin-3 (Gal3) is both endogenously expressed as well as released by microglia in the brain in pathological conditions (Garcia-Revilla et al., 2022), particularly in Alzheimer’s disease (AD) (Boza-Serrano et al., 2019). Its dual presence inside cells and in the extracellular space suggests that Gal3 may exert distinct, context-dependent effects on microglial function. Extracellular Gal3 can act in a paracrine or autocrine manner to influence neighboring cells as well as itself (Burguillos et al., 2015), while intracellular Gal3 contributes to cell-autonomous processes. Interestingly, homeostatic microglia barely express Gal3 at the transcriptomic level (Garcia-Revilla et al., 2022; Boza-Serrano et al., 2019). To investigate these functions, we generated Gal3-deficient BV2 microglia using the Alt-R CRISPR-Cas9 system (Integrated DNA Technologies). BV2 cell line is a murine microglial cell line with basal Gal3 expression (Figures 1a,b). We successfully generated several microglial BV2 cell line clones showing no levels of endogenous Gal3 at transcript and protein levels (Figures 1a,b). Clone 3 was selected for all further experiments (referred to as Gal3KO). In order to explore if other galectins were affected by this deletion, relative transcript expression was analysed (Figure 1c). Based on previously published transcriptomic data in microglia, galectins −1, −8 and −9 were studied (Krasemann et al., 2017). Besides Gal3, no other measured galectin showed any significant change (Figure 1c), suggesting no compensatory mechanism at least through another galactoside-binding protein. Curiously, we detected that Gal3bp, known to be a ligand for several galectins in the extracellular matrix, including Gal3, was also decreased in Gal3KO cells, indicating a transcriptional regulation driven by Gal3.

### Endogenous galectin-3 alters microglial area, mitochondrial content, and motility

**Figure 2:**
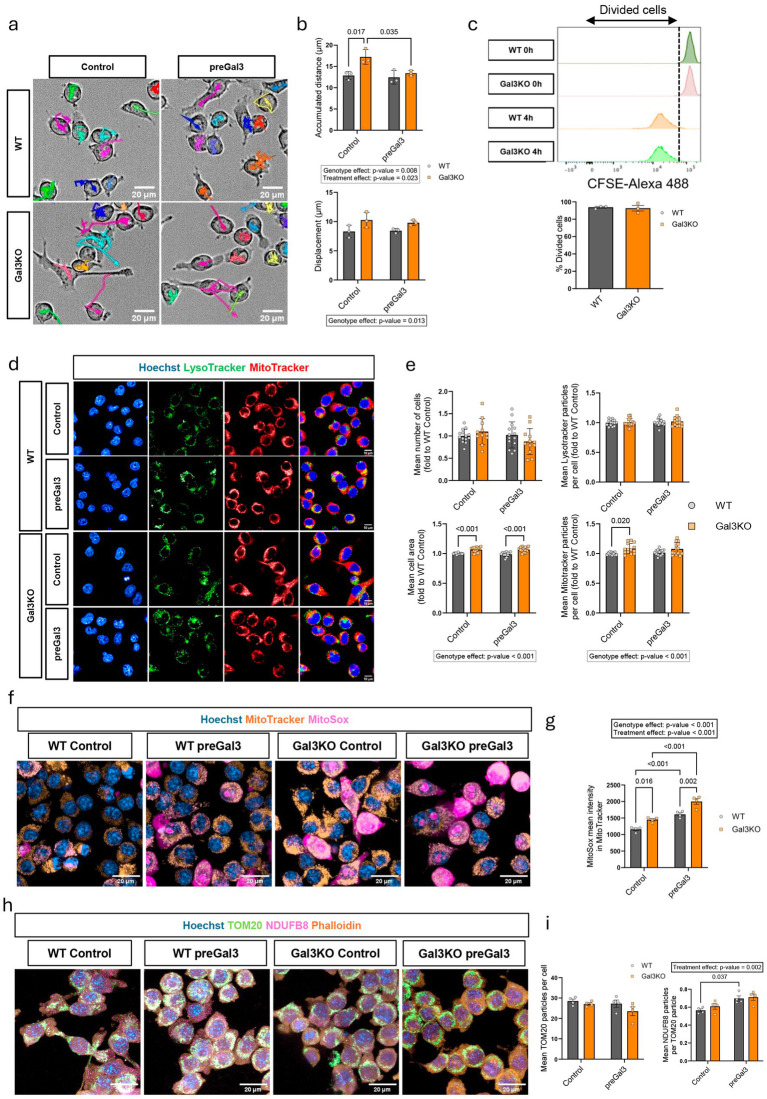
Analysis of microglial motility, organelle content, proliferation and mitochondrial activation under endogenous and exogenous galectin-3 modulation. **(a)** Representative bright field images of BV2 WT and Gal3KO cells treated with exogenous Gal3 for 24 h (preGal3) with individual cell motility throughout the experiment (colored arrows). Scale bars, 20 μm. **(b)** Quantification of accumulated distance and displacement of BV2 cells in cell motility experiment. *n* = 3 per group. **(c)** CFSE cell division assay quantification using flow cytometry, with percentage of divided cells per group. *n* = 3 per group. **(d)** Representative live-cell fluorescence imaging of BV2 cells showing nuclei (Hoechst), lysosomes (LysoTracker) and mitochondria (MitoTracker). Scale bars, 10 μm. **(e)** Quantification of cell number, area, lysosomal content and mitochondrial content. *n* > 10 per group. **(f)** Representative live-cell images of BV2 cells showing mitochondrial superoxide (MitoSox), nuclei and mitochondria. Scale bars, 20 μm. **(g)** Quantification of MitoSox intensity within MitoTracker particles. *n* = 4 per group. **(h)** Representative immunofluorescence images of mitochondria (TOM20) and mitochondrial complex I (NDUFB8). Scale bars, 20 μm. **(i)** Quantification of total mitochondrial and complex I content within mitochondria. *n* = 4 per group. Data are shown as individual replicates (each experimental replicate corresponds to an independent culture performed from a different cell passage) with mean ±SEM. In **(b,e,g,i)**, two-way ANOVA with Tukey’s multiple comparisons was performed and significant ANOVA differences are shown. In c, unpaired parametric t-test was used. *p*-values are expressed with 3 decimals.

Microglia are highly mobile cells, and their proper mitochondrial and lysosomal function are key elements of neurodegeneration (Agrawal and Jha, 2020; Nixon, 2017; Lively and Schlichter, 2013). Therefore, to evaluate the differential involvement of endogenous and exogenous Gal3 in these microglial functions, we exposed WT and Gal3KO BV2 cells with 1 μM of exogenous Gal3 (preGal3, Supplementary Figure 1a). Cells were first tracked for 6 h using the Operetta CLS high-content imaging system (Revvity) (Figure 2a). Gal3KO cells displayed significantly greater accumulated traveled distance compared to WT counterparts, whereas preGal3 treatment decreased the motility to WT levels, effectively equalizing both groups (Figure 2b). To further investigate if exogenous Gal3 yielded an effect on cell motility, we performed a scratch in WT cells and determined that exogenous Gal3 at the time of scratch rather than before, was detrimental for microglial motility after 6 and 24 h (Supplementary Figures 1b–d), anticipating a fine tuning anti-migratory effect for Gal3.

Microglial proliferation is considered a signal for microglial activation and response to brain damage (Hammond et al., 2018). Therefore, to test whether Gal3 genetic deletion affected proliferation and cell division, we performed a CFSE assay, where CFSE fluorescent dye is incorporated to the cell and its content is diluted division after division. No significant differences were observed in the proportion of divided cells after 4 h, when the majority of cells had already divided in both genotypes (Figure 2c). Furthermore, we analyzed cell cycle stage by measuring nuclear DNA content based on the summed Hoechst fluorescence intensity within each nucleus (Olofsson et al., 2021). As observed, most of the cells present an intensity related to G1 phase which approximately doubles in phase G2/M, whereas intermediate intensities were assigned to phase S (Supplementary Figures 2a,b). Again, no difference was found between the genotypes in G2/M phase proportion of cells (Supplementary Figure 2c). Thus, supporting no difference in cell cycle and division rate.

Since microglia’s motility and tropism toward damaged brain areas are highly energy-consuming mechanisms, we evaluated the mitochondrial status after Gal3 deletion and exogenous exposure. Utilizing the Operetta CLS high-content imaging system, we quantified cell number, cell area, lysosomal content, and mitochondrial content after 24 h (Figure 2d). Notably, Gal3KO resulted in a highly significant genotype effect, with increased cell area and MitoTracker particle content, while preGal3 equalized WT and Gal3KO cells in terms of mitochondrial content (Figure 2e). Despite no change in cell area being detected after 24 h, we observed that exogenous Gal3 exposures on WT cells promoted a time-dependent altered microglial morphology over time, from a more rounded to a more elongated shape (Supplementary Figure 2d). In contrast, the total number of cells and the number of lysosome particles remained unaffected (Figure 2d). These findings suggest that Gal3KO cells may have an increased energy expenditure. To further investigate this hypothesis, we tested the production of superoxide with the fluorescent dye MitoSox (Figures 2f,g). Interestingly, Gal3KO cell had a significant overproduction of superoxide while exogenous Gal3 addition also increased the production of superoxide species (Figure 2g). Since superoxide production is mainly produced by complex I activity in the electron transport chain pathway (Kussmaul and Hirst, 2006), we next assessed the levels of mitochondrial external membrane TOM20 and complex I subunit NDUFB8 (Figures 2h,i). Complex I subunit levels correlated with superoxide production, thus supporting a change in this complex as the main source of superoxide production and explaining the observed MitoSox elevation (Figure 2i). However, the number of TOM20 mitochondria remained stable after preGal3 treatment and Gal3 deletion. This is intriguing as it does not correlate with the levels of MitoTracker, MitoSox or NDUFB8. MitoTracker accumulation initially depends on mitochondrial membrane potential, as well as MitoSox depends on complex I activity, while TOM20 is a receptor protein in the mitochondrial outer membrane not related to the oxidative phosphorylation pathway.

This led us to conclude that endogenous Gal3 is important on microglial metabolism and motility without affecting their division, while exogenous Gal3 mainly impacts migration. An increase mitochondrial oxidative phosphorylation pathway is suggested to occur by both Gal3 deletion and Gal3 exogenous stimulation, showing a dual role for Gal3, while total mitochondrial content may remain stable as suggested by levels of TOM20.

### Differential regulation of microglial uptake pathways by galectin-3

**Figure 3:**
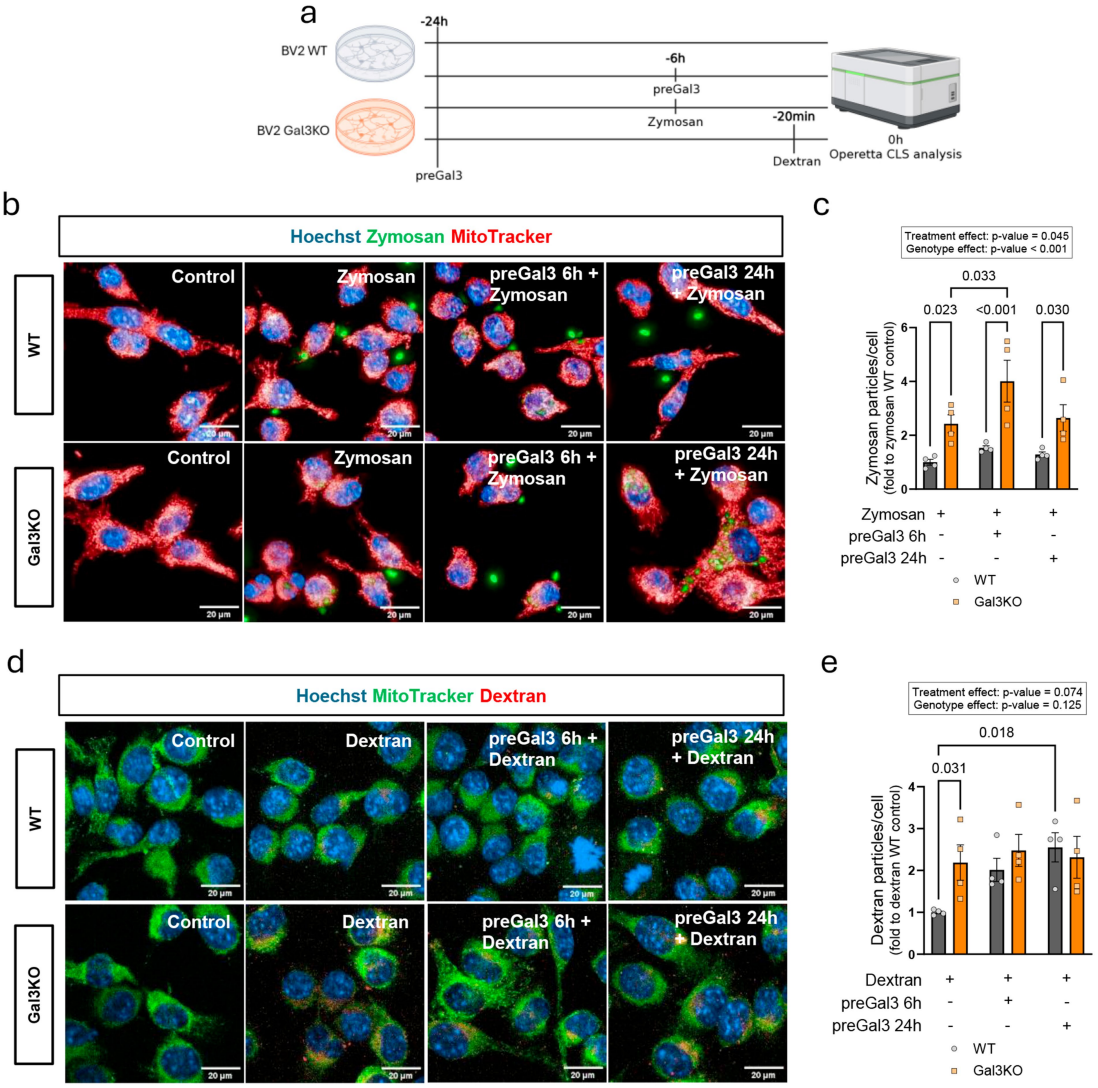
Effects of endogenous and exogenous galectin-3 modulation on microglial phagocytosis and endocytosis. **(a)** Schematic experimental outline of phagocytosis and endocytosis experiments of BV2 WT and Gal3KO cells after pretreatment with Gal3 (preGal3) for 24 or 6 h (created with biorender.com). **(b)** Representative fluorescence imaging of cells treated with zymosan particles. **(c)** Number of zymosan particles per cell quantification. **(d)** Representative fluorescence imaging of cells treated with dextran particles. **(e)** Number of dextran particles per cell quantification. Data are shown as individual replicates with mean ± SEM (each experimental replicate corresponds to an independent culture performed from a different cell passage). Scale bars, 20 μm, *n* = 4 per group. In **(c,e)**, two-way ANOVA with Tukey’s multiple comparisons was performed and significant ANOVA differences are shown. *p*-values are expressed with 3 decimals.

Another key feature of microglial cells is the surveillance of the extracellular matrix and the clearance of debris and pathogens. Thus, we next assessed phagocytosis and endocytosis function in WT and Gal3KO BV2 cells, as well as the impact of preGal3 treatment (Figure 3a). For this purpose, we measured the uptake of zymosan and dextran particles, which have been shown to be linked to phagocytosis (Venkatachalam et al., 2020) and endocytosis (Li et al., 2015) pathways, respectively. Here, exogenous Gal3 was added before (preGal3 24 h), or at the same time as zymosan (coincubation 6 h) or 6 h prior dextran (preGal3 6 h); while MitoTracker was used as a counterstain for cytosolic content. Phagocytosis, measured by zymosan internalization, was markedly enhanced in Gal3KO cells, however, a mild synergistic treatment effect of preGal3 appears, being more intense when Gal3 was added along with zymosan particles (Figures 3b,c). Again, a discrepancy emerges between the roles of endogenous and exogenous Gal3, suggesting a dual role. Endocytosis, measured as dextran uptake, was also increased under basal conditions in Gal3KO cells, while exogenous Gal3 had no effect on Gal3KO cells but further increased endocytosis in WT cells (Figures 3d,e). These results indicate that endogenous Gal3 constrains phagocytic and endocytic activity, as Gal3KO cells present increased levels of both mechanisms. The role of exogenous Gal3 is intriguing, as it limitedly increases phagocytic activity independently of endogenous Gal3 presence, resembling its role as opsonin (Karlsson et al., 2009). In contrast, exogenous Gal3 promotes endocytosis exclusively in WT cells but not in Gal3KO cells, arising the idea that extracellular/intracellular Gal3 balance is determinant in this pathway.

### Galectin-3 controls microglia activation through immune receptor modulation and TNFα secretion

**Figure 4:**
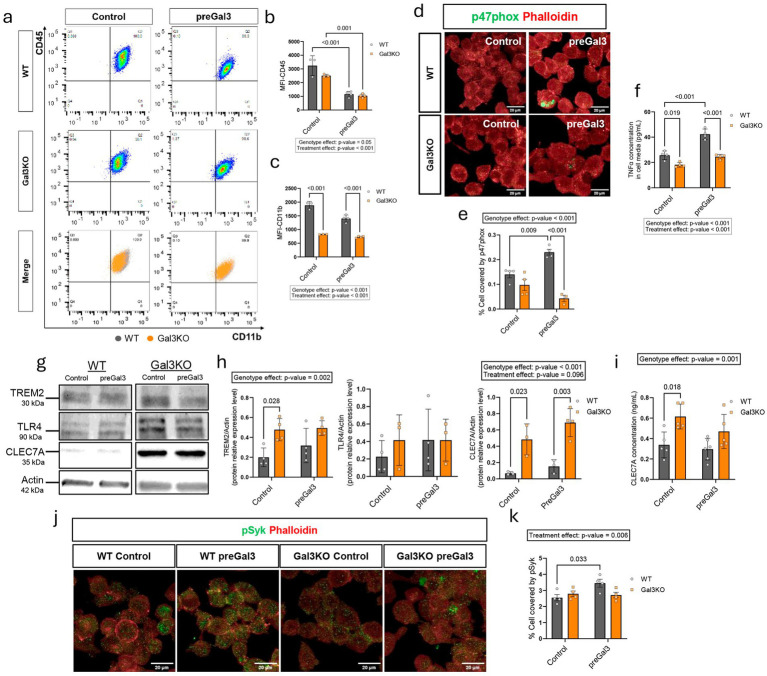
Analysis of microglial activation markers and inflammatory pathways under endogenous and exogenous galectin-3 modulation. **(a)** Flow cytometry analysis of BV2 WT and Gal3KO cells after Gal3 24 h pretreatment (preGal3) by CD11b and CD45 surface markers. 20,000 events were analyzed. Gating strategy is shown in Supplementary Figure 3. *n* = 3 per Control group and *n* = 4 per preGal3 group. **(b)** Quantification of median fluorescence intensity (MFI) of CD45 surface marker. **(c)** Quantification of MFI of CD11b surface marker. **(d)** Representative immunofluorescence images of NOX2 subunit p47phox. Scale bars, 20 μm. **(e)** Quantification of total p47phox content within BV2 cells. *n* = 4 per group. **(f)** TNFα concentration in cell medium measured by Mesoscale using a V-PLEX panel. TNFα was detected within the detection range consistently throughout all samples. *n* = 4 per group. **(g)** Representative Western blot images of BV2 WT and Gal3KO protein lysates. **(h)** Western blot quantification of TREM2, TLR4 and Clec7a expressed as the ratio divided by Actin. *n* = 3–4 per group. **(i)** Clec7a ELISA quantification. *n* = 5 per group. **(j)** Representative immunofluorescence images of phosphorylated Syk (pSyk). Scale bars, 20 μm. **(k)** Quantification of total pSyk content within BV2 cells. *n* = 4 per group. Data are shown as individual replicates with mean ±SEM (each experimental replicate corresponds to an independent culture performed from a different cell passage). In **(b,c,e,f,h,i,k)**, two-way ANOVA with Tukey’s multiple comparisons was performed and significant ANOVA differences are shown. *p*-values are expressed with 3 decimals.

Next, we proposed to look into general immune features of microglial cells upon Gal3 deletion and exogenous presence. CD11b and CD45 are common surface markers used to define the microglial population in mouse models and human brain (Kettenmann et al., 2011). Thus, we performed flow cytometry analysis of CD11b and CD45 (Masliah et al., 1991; Czirr et al., 2017) in WT and Gal3KO cells exposed to preGal3 for 24 h (Figure 4a; Supplementary Figures 3a,b). Median fluorescence intensity (MFI) analysis revealed that CD45 surface levels were slightly reduced following Gal3 pretreatment in both genotypes (Figure 4b). CD11b levels also decreased after preGal3 treatment in WT cells, but, strikingly, Gal3 deletion produced a robust reduction in CD11b levels (Figure 4c), indicating a critical role of endogenous Gal3 in regulating this marker. Next, to assess the contribution of Gal3 to microglial inflammatory activity, we measured the NOX2 subunit p47phox. NOX2 is a hallmark of pro-inflammatory microglial activation and is capable of generating superoxide species (Qin et al., 2004). Interestingly, p47phox levels increased in WT cells after Gal3 exogenous exposure, however, Gal3 genetic deletion diminished its expression (Figures 4d,e). While p47phox increased levels are suggestive of a pro-inflammatory status of the cell (Yamagata et al., 2014), p47phox must be recruited by p22phox subunit and extensively phosphorylated during NOX2 complex activation (Paclet et al., 2022). Similarly, p22phox showed a detrimental effect following Gal3 genetic deletion, but no effect for exogenous Gal3 (Supplementary Figures 3c,d), however, colocalization of both markers showed no difference, meaning that no differential activation of NOX2 complex was produced (Supplementary Figure 3d). In line with p47phox, proinflammatory cytokine secretion was next evaluated. Similarly, preGal3 treatment in WT cells for 24 h increased TNFα release, as measured by TNFα concentration in the cell media, supporting both autocrine and paracrine inflammatory roles for Gal3 (Figure 4f). In contrast, Gal3KO cells exhibited reduced TNFα secretion under both basal and preGal3 conditions (Figure 4f). Other measured cytokines were not consistently within detection range. Curiously, we also detected that WT BV2 can secrete TNFα in response of both Gal3 or its galectin relative Gal1 (Supplementary Figure 4a). Thus, we proposed to verify whether Gal1 TNFα release was dependent on endogenous Gal3. To verify the unique autocrine/paracrine effects of Gal3, we exposed BV2 cells to increasing Gal1 dose. Both WT and Gal3KO cells responded in similar ways to Gal1 exposure in terms of *Tnfa* gene expression (Supplementary Figure 4b). Similarly, no genotype effect was observed for Gal1 induced *Il1b* gene expression (Supplementary Figure 4b). Indicating that the genotype effect for endogenous Gal3 in TNFα is only detected when exogenous Gal3 is the stimuli, but not other galectin, thus confirming a paracrine effect of Gal3 specifically.

Since microglial Gal3 has been implicated in multiple aspects of AD pathogenesis (Garcia-Revilla et al., 2022; Boza-Serrano et al., 2019; Tao et al., 2020; Boza-Serrano et al., 2022), we next investigated its relationship with microglial receptors that play central roles in microglial phenotype regulation and AD progression: TREM2, TLR4, and Clec7a (Hansen et al., 2018; Valiukas et al., 2025; Krasemann et al., 2017; Keren-Shaul et al., 2017; Huang et al., 2021; Miron et al., 2018; Roy et al., 2020). Western blot analysis demonstrated a clear genotype effect, with Gal3KO cells showing elevated TREM2 expression and, most strikingly, a dramatic increase in Clec7a levels compared to WT (Figures 4g,h). Interestingly, TLR4 remained unchanged, suggesting that Gal3 selectively modulates a subset of AD-associated receptors. To further confirm the robust Clec7a upregulation, we performed an ELISA assay, which validated the strong increase in Clec7a content in Gal3KO cells (Figure 4i). Both Clec7a and TREM2 converge into the phosphorylation of Syk (pSyk) in their downstream activation pathway (Wang et al., 2022; Nascimento et al., 2025), thus we stained the BV2 cells to assess the levels of pSyk (Figure 4j). WT and Gal3KO cells present similar basal levels of pSyk, increasing after preGal3 treatment only in WT but not in Gal3KO cells (Figure 4k). This means that the increased levels of TREM2 and Clec7a provoked by Gal3 deletion do not correlate with downstream Syk phosphorylation.

Overall, these findings suggest that endogenous and exogenous Gal3 differentially shape microglial receptor regulation and inflammatory output. Exogenous Gal3 appears to drive CD45 internalization and promote autocrine and paracrine proinflammatory responses, whereas endogenous Gal3 is required to maintain CD11b surface expression and support robust cytokine release. The loss of Gal3, therefore, shifts microglia toward a state with reduced CD11b availability but increased TREM2 and Clec7a receptors, but only WT cells are responsive to exogenous Gal3 addition in terms of pathway activation as shown by Syk phosphorylation and TNFα secretion. These data highlights distinct mechanisms by which endogenous versus exogenous Gal3 fine-tunes microglial activation.

### Galectin-3 deletion selectively upregulates Clec7a in Alzheimer’s disease mouse model without affecting microglia recruitment to amyloid plaque

**Figure 5:**
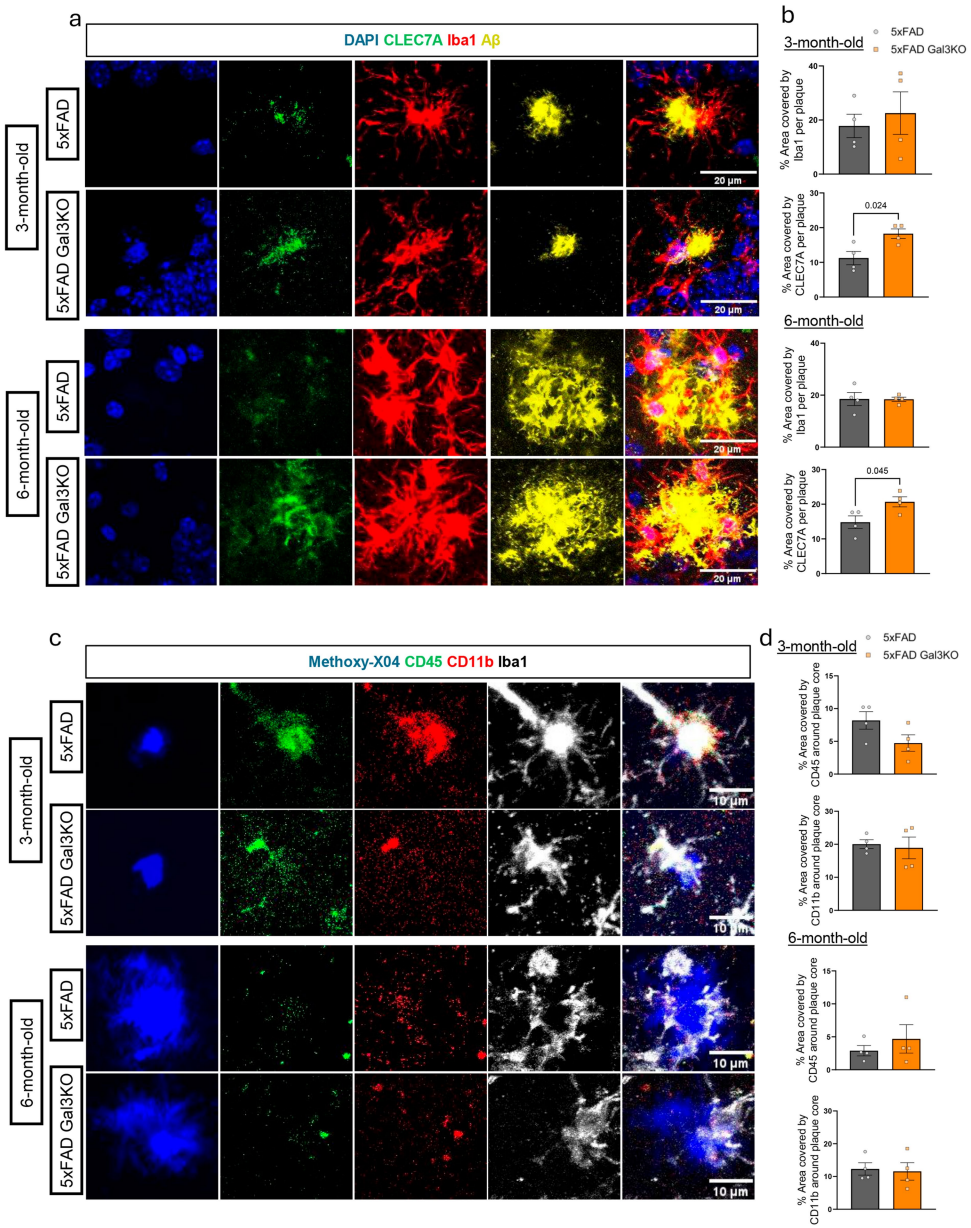
*In vivo* assessment of microglial plaque recruitment and receptor activation in 5xFAD mouse model after Gal3 deletion. **(a)** Representative immunostaining of Iba1 and Clec7a around Aβ plaques (MOAB2) in 5xFAD and 5xFAD Gal3KO mouse brains. Scale bars, 20 μm. **(b)** Quantification of percentage of the peri-plaque area covered by Iba1 and Clec7a in 3- and 6-month-old female mice. *n* = 4 per group. **(c)** Representative immunostaining of CD45 and CD11b around plaque cores (Methoxy-X04). Scale bars, 10 μm. **(d)** Quantification of percentage of the peri-core area covered by CD45 and CD11b. *n* = 4 per group. Data are shown as individual replicates with mean ±SEM (each experimental replicate corresponds to a different mouse). A minimum of 20 plaques per mice were analyzed in the dentate gyrus. In **(b,d)**, unpaired parametric *t*-test was used. *p*-values are expressed with 3 decimals.

Our group has previously characterized a reduction of plaque burden in the 5xFAD model following Gal3 genetic deletion at 6- and 18-month-old mice (Boza-Serrano et al., 2019). In this study, a Gal3-TREM2 interaction was described, in line with our *in vitro* results. Thus, we then tested if the observed changes in CD45, CD11b and Clec7a after Gal3 genetic deletion were observed *in vivo*. In this line, we used 3- and 6-month-old female 5xFAD and Gal3KO-5xFAD mice brains (Figures 5a,c). Firstly, microglia recruitment, measured as Iba1-positive area around the plaque was similar between genotypes at both age timepoints (Figures 5a,b). Next, we assessed Clec7a peri-plaque levels. In this case, Clec7a coverage per amyloid plaque was significantly greater in Gal3KO mice at both 3 and 6 months of age (Figures 5a,b), indicating that the Clec7a upregulation observed *in vitro* in the absence of Gal3 is maintained during AD pathology. Similarly to Iba1, CD11b and CD45 showed no differences between genotypes at any timepoint (Figures 5c,d), thus supporting no differences in microgliosis and no effect of Gal3 toward these receptors in the context of AD.

Taken together, these results identify Gal3 as a negative regulator of Clec7a *in vivo*. This connection links Gal3 to one of the most relevant AD-associated microglial receptors and suggests that Gal3 influences microglial phenotype both under basal conditions and in the context of neurodegeneration.

## Discussion

Galectin-3 (Gal3) is a pleiotropic lectin commonly produced by immune cells with high impact on neurodegenerative diseases (Garcia-Revilla et al., 2022). Our group has previously demonstrated a critical role for microglial Gal3 in AD interacting with the microglial receptor TREM2 (Boza-Serrano et al., 2019) and TLR4 (Burguillos et al., 2015), as well as being an important molecule in protective immune priming in AD (Yang et al., 2023). The role of Gal3 in AD mouse models is detrimental, as the absence of Gal3 improves the disease outcome in mice. Importantly, increased Gal3 levels have also been observed in AD patients’ brains and CSF (Boza-Serrano et al., 2019; Boza-Serrano et al., 2022), demonstrating an interesting correlation with the human pathology. However, the cellular roles of Gal3 within the microglial cell have not been explored yet, and the complexity in determining its precise roles is a limitation for *in vivo* studies. For instance, we have demonstrated that Gal3 can be secreted by microglia under certain circumstances, playing roles such as opsonization of damaged neurons in PD models and further phagocytosis (Garcia-Revilla et al., 2024), but whether only extracellular or intracellular Gal3 plays a role in phagocytosis is not known. Here, we further explore the roles of microglial Gal3 by creating a new and stable Gal3KO microglial BV2 cell line. Our findings demonstrate the variety roles that Gal3 plays in microglia, placing Gal3 as a critical molecular modulator of microglial activity, controlling not only AD-related activation but also homeostatic functions. Rather than acting solely as a pro-inflammatory mediator, Gal3 emerges as a fine tuning of microglia homeostatic and non-homeostatic activity. This duality challenges the traditional view of Gal3 and highlights its central role in shaping microglial responses to pathology.

Gal3 genetic removal in BV2 microglial cell line promoted homeostatic changes affecting basic cellular processes such as mitochondrial modulation, increased cell size, and enhanced motility. The elevation in mitochondrial oxidative phosphorylation pathway may be driven by the increased motility and cell size, both of which demand higher energy production. However, the role of Gal3 in mitochondrial homeostasis remains debated. In pancreatic cell lines, Gal3 silencing leads to the accumulation of dysfunctional mitochondria (Coppin et al., 2020), and Gal3 has been reported as a mitochondria-associated membranes (MAM) protein that preserves mitochondrial network integrity and repair in endothelial cells (Coppin et al., 2020). In contrast, Gal3 has been shown to inhibit mitochondrial complex activity in T cells (Wang et al., 2023). In our model, Gal3 deletion in BV2 microglia appears to exert a neutral or even positive effect on basic cellular homeostasis, as Gal3KO cells showed no differences in proliferation as measured by CFSE intensity assay or nuclear DNA content analysis. Nonetheless, notable differences emerged in mitochondrial status. Gal3KO BV2 cells exhibited higher levels of MitoTracker, MitoSox, and complex I subunit expression, while the total mitochondrial content remained unchanged. This pattern suggests an increased oxidative phosphorylation pathway driven by higher complex I abundance and activity, supported by elevated NDUFB8 levels, increased accumulation of membrane potential-sensitive MitoTracker dye, and enhanced superoxide production. Given that superoxide production primarily relies on complex I activity, we conclude that BV2 Gal3KO cells present higher basal levels of oxidative phosphorylation, in line with what has been described in T-cells (Wang et al., 2023). Importantly, this increase is not attributable to a greater number of mitochondria, as indicated by stable TOM20 levels. Strikingly, exogenous Gal3 addition also enhanced oxidative phosphorylation in both genotypes. One contributing factor may be the microglial response to Gal3, which we later show activates WT BV2 cells toward a proinflammatory phenotype marked by elevated TNFα and NOX2 levels. However, because exogenous Gal3 also increased superoxide production in Gal3KO cells (which showed a much lower proinflammatory response) the inflammatory phenotype alone cannot fully explain the metabolic effect. Interestingly, Zhou et al. demonstrated that exogenous Gal3 enhances endothelial cell metabolism by increasing both oxygen consumption and glycolysis through Jag1 binding and Notch pathway regulation (Zhou et al., 2023). Whether a similar mechanism operates in microglia remains unknown, but our results point to a direct effect of exogenous Gal3 on microglial metabolism.

Beyond metabolic functions, Gal3 may also affect other homeostatic functions of microglia. Indeed, the addition of Gal3 was shown to act as a migratory-tunning molecule based on our data. Previous reports have stated a pro-migratory effect for exogenous Gal3 (Wesley et al., 2013). Here, we describe a different effect, in live-cell tracking addition of exogenous Gal3 resulted in reduced motility and reduced wound healing when produced 24 h after Gal3 addition, while when Gal3 was added at the same time of wound healing, it increased migration. Therefore, the migratory effects of Gal3 may be time-limited, potentially leading to desensitization after prolonged exogenous Gal3 exposure. Indeed, Gal3KO microglia displayed markedly improved uptake functions both in phagocytosis and endocytosis which could be favored by enhanced motility and metabolic capacity. This coupling of mitochondrial content with phagocytic performance is consistent with the notion that energy availability is a key determinant of microglial efficiency (Coppin et al., 2020; Sadeghdoust et al., 2024; Jung et al., 2025).

In parallel, Gal3 genetic deletion altered phenotypic markers and inflammatory responses. CD11b and CD45 are common surface markers used for microglia detection/isolation within the brain (Volden et al., 2015). CD11b, also known as integrin αMβ2 or ITGAM, is wide express in microglia across all brain regions (Doorn et al., 2015). CD11b increased expression has been shown in microglia after bacterial infection (Hoogland et al., 2015) and neurodegenerative conditions (Hou et al., 2020), but is thought to be a immunomodulatory receptor acting on TLR4 pathway (Yang et al., 2018). Interestingly, gene variations in ITGAM gene have been related to AD (Salih et al., 2019). Our data demonstrate that Gal3KO BV2 cells showed lower CD11b surface expression, and lower TNFα release, indicating a shift away from detrimental neuroinflammatory functions (Hansen et al., 2018; Czirr et al., 2017; Plantone et al., 2023). This is reflected in the levels of the NOX2 subunit p47phox, which increases after exogenous Gal3 treatment in WT cells but not in Gal3KO cells, where both basal and treated levels remain lower. The p22phox subunit is also reduced in Gal3KO cells. However, our experimental conditions did not lead to increased assembly of these subunits, which should be examined under alternative settings.

Since major recently described neuroinflammatory microglial phenotypes (such as DAM or MGnD) typically downregulate homeostatic genes and upregulate Gal3 itself (Garcia-Revilla et al., 2022; Wei and Li, 2022), our findings suggest that the absence of Gal3 reinforces a unique microglial phenotype that may act as a buffer against excessive inflammation. Beyond transcriptomic regulation, we have previously demonstrated the ability of microglial cells to release Gal3 protein to the extracellular space in response to both LPS (Burguillos et al., 2015) and fibrillar Aβ (Boza-Serrano et al., 2019) and confirmed its importance for full-blown microglial activation (Burguillos et al., 2015). Although we are aware of the distinct acute versus chronic roles of Gal3, the present study focused on long-term exogenous Gal3 exposure to better mimic the sustained neuroinflammatory environment characteristic of chronic neurodegenerative diseases such as AD (Hansen et al., 2018). The autocrine/paracrine effect of Gal3, acting both endogenously and exogenously, adds complexity to the readout of the results. To overcome that limitation, we have also proved that Gal3KO does not alter the inflammatory effects of another galectin analog, Gal1, thus meaning that Gal3 deletion affects the response of Gal3 exogenous addition exclusively suggesting a paracrine effect. Nevertheless, some effects could be directly explained to exogenous Gal3, as it promoted a marked decline in CD45 surface expression both in WT and Gal3KO cells. Interestingly, exogenous Gal3 has been previously reported to act stabilizing membrane CD45 and triggering apoptosis (Farhadi et al., 2021) in T-cells, which was not observed in our model. In microglia, intermediate levels of surface CD45 are observed, and is thought to mediate phagocytic pathways, including amyloid processing (Rangaraju et al., 2018) while increasing DAM markers such as TREM2 or Clec7a. Interestingly, we observed reduced surface CD45 expression with exogenous Gal3 addition, while not affecting TREM2 or Clec7a levels. This was further tested in the 5xFAD mice where Gal3 deletion did not affect CD45, CD11b or Iba1 levels around plaques, confirming no modification of microgliosis in our model. Our *in vitro* data also opens the possibility for a CD45 internalization following *in vitro* exogenous Gal3 stimuli, which should be further assessed in the *in vivo* model.

Similarly, we have also assessed phagocytosis through zymosan uptake. Zymosan is a known TLR2 and Clec7a ligand (Dillon et al., 2006), and the effect of Gal3 in this process was dual. On one hand, exogenous Gal3 enhanced zymosan uptake; however, Gal3 genetic deletion showed a higher predisposition for zymosan uptake, while also exhibiting a mild response to exogenous Gal3. This duality may be explained by increased basal levels of TLR2 and/or Clec7a in response to Gal3 genetic deletion. While Gal3 is known to be a TLR4 ligand (Burguillos et al., 2015), no direct binding to TLR2 has been proposed, however, Gal3 is known to be a Clec7a partner (Esteban et al., 2011) enhancing Clec7a recognition of fungal and bacterial polysaccharides. This was confirmed by the assessment of Clec7a protein levels in BV2 cells which also showed a dual response, mildly increasing by exogenous Gal3, and highly increasing by Gal3 genetic deletion. Noteworthy, Clec7a has been implicated in AD through its ability to bind Aβ and initiate Syk/NF-κB-dependent responses (Wang et al., 2022). Moreover, Clec7a is a recognized DAM marker, known to be expressed by plaque-surrounding microglia (Garcia-Revilla et al., 2022; Wei and Li, 2022). In Gal3KO 5xFAD mice, plaque burden is reduced in 6 months-old mice as we have previously demonstrated (Boza-Serrano et al., 2019). We have now studied the levels of Clec7a in this model at 3- and 6-month-old where Aβ plaques are already present (Lee et al., 2024). Clec7a coverage was significantly increased around plaques, confirming the *in vivo* translation of our previous results. Interestingly, TREM2 showed a similar genotypic pattern but remained insensible to exogenous Gal3 in our *in vitro* assay, while it is known to increase in 5xFAD mice. Importantly, the levels of pSyk, a common downstream mediator of both TREM2 and Clec7a pathways reflected a different pattern in BV2 cells. pSyk was elevated after exogenous Gal3 addition only in WT but not Gal3KO cells. This first confirms the effect on Gal3 as a TREM2/Clec7a ligand, and secondly, demonstrates the importance of endogenous Gal3 in sustaining this effect. This complex Gal3-Clec7a regulatory axis may represent a critical mechanism through which microglia balance amyloid clearance with inflammatory activation, and its modulation could have direct consequences for AD progression (Nascimento et al., 2025). The double exogenous/endogenous Gal3 regulation together with direct Gal3-Clec7a binding, suggests a negative feedback regulation of Clec7a expression mediated by Gal3 interaction. Therefore, while exogenous Gal3 clearly influences microglial function through extracellular mechanisms, it may also be partially internalized, thereby exerting additional intracellular effects.

These findings carry important therapeutic implications. Inhibiting Gal3 may reduce chronic inflammation and balance phagocytosis/endocytosis without hampering the expression of TREM2 and Clec7a which are thought to have a positive effect in some diseases such as AD (Wang et al., 2022). Both exogenous presence and complete deletion of Gal3 appear to push microglia away from equilibrium, suggesting that therapeutic strategies should aim to fine-tune rather than abolish its activity. Selective targeting extracellular Gal3 may be a good alternative for restoring microglial balance in AD. Our results reveal Gal3 as a multifaceted regulator of microglial homeostasis, phagocytosis, receptor expression, and inflammatory output. By acting as a homeostatic checkpoint, Gal3 shapes the trajectory of microglial responses to amyloid pathology, positioning it as a potential but complex therapeutic target in Alzheimer’s disease.

## Data Availability

The original contributions presented in the study are included in the article/Supplementary material, further inquiries can be directed to the corresponding author.
