# Supplementary material for: Galectin-3 shapes microglial phenotype through endogenous and exogenous mechanisms
Source: Front Cell Neurosci. 2025 Dec 18;19:1729776. doi: 10.3389/fncel.2025.1729776 (PMC12756361; doi:10.3389/fncel.2025.1729776)
Supplement: Supplementary file 1 [file Table_1.docx]

**Galectin-3 shapes microglial phenotype through endogenous and exogenous mechanisms**

Lluís Camprubí-Ferrer^1*^, Yiyi Yang^1^, Rosalía Fernández-Calle^1^, Antonio Boza-Serrano^1,2,3^, Juan García-Revilla^1,2, 3#^, Javier Frontiñán-Rubio^4^^#^, Tomas Deierborg^1#^

**Supporting information**


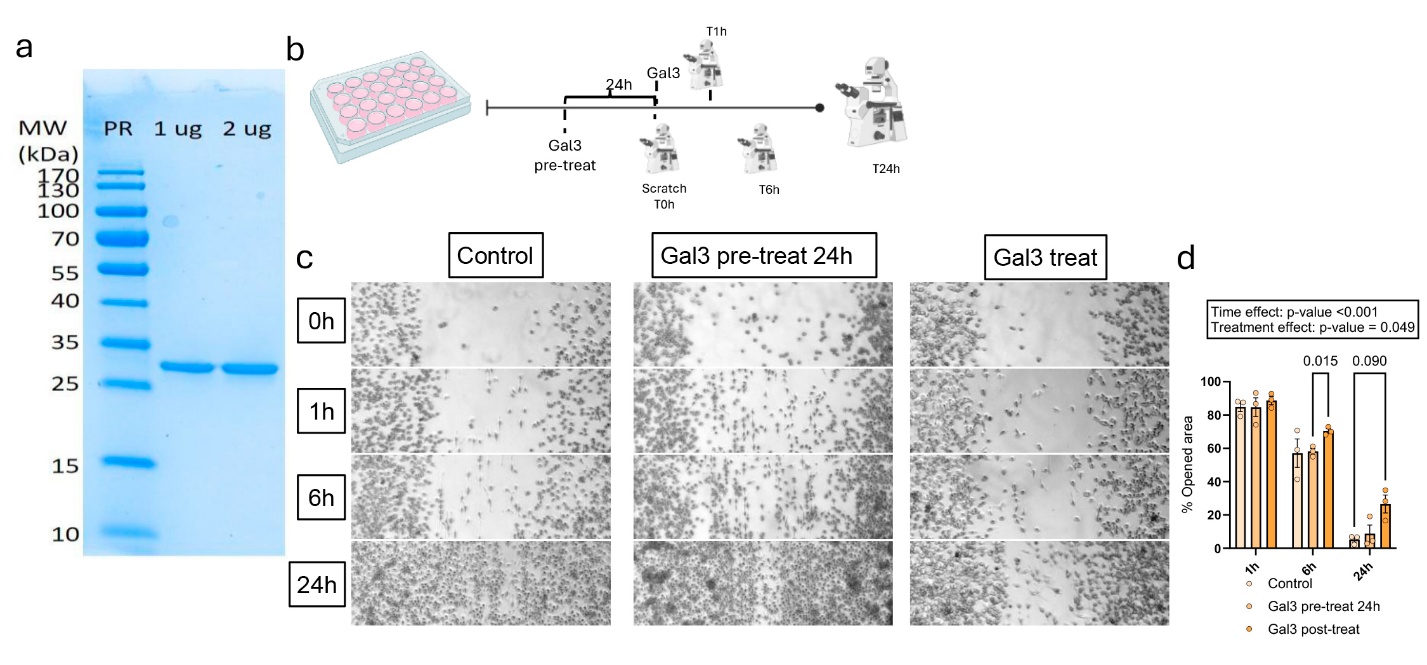


**Suppl. Fig. 1. Recombinant human galectin-3 gel staining and BV2 WT and Gal3KO cells scratch assay.**

**a** 1 μg and 2 μg of human Gal3 on a SDS-PAGE gel stained with Bio-Safe Coomassie (Bio-rad). **b** Schematic experimental outline of scratch assay of BV2 WT after pretreatment and treatment with Gal3 (created with biorender.com). **c** Representative bright field images of BV2 cells at different timepoints of scratch assay. **d** Cellular movement after scratching at different timepoints, measures by the percentage of the wound still opened. In d, two-way ANOVA with Tukey’s multiple comparisons was performed and significant ANOVA differences are shown. P-values are expressed with 3 decimals.


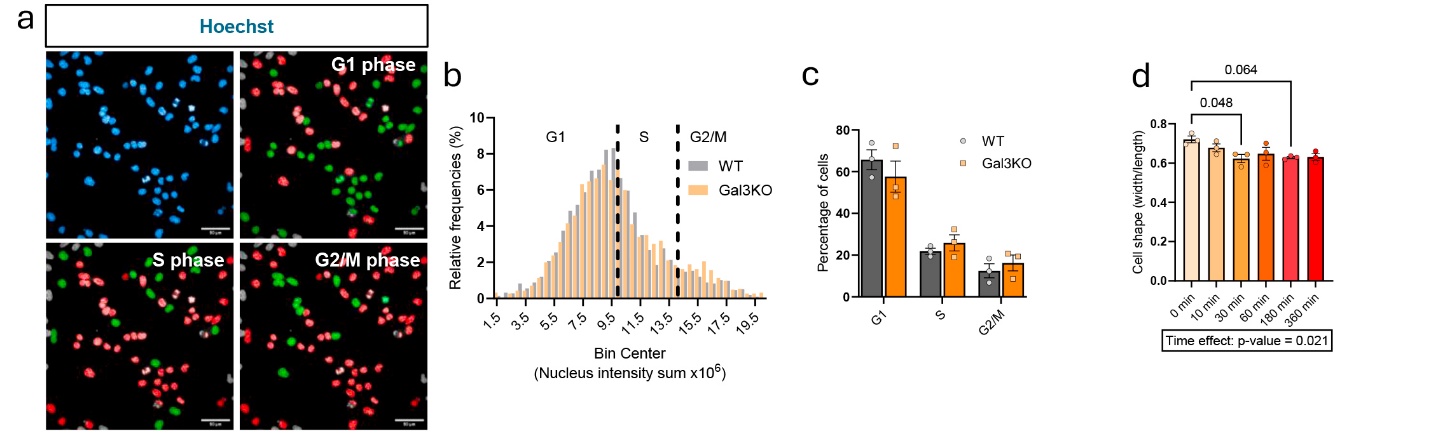


**Suppl. Fig. 2. Cell division phase analysis on BV2 WT and Gal3KO cells and morphology effects of acute Gal3 treatment on BV2 WT cells.**

**a** Live-cell fluorescence images of BV2 cells stained with Hoechst, with population analysis showing cells in G1, S, and G2/M phases (green indicates the identified cell state). Scale bars, 50 µm, n=3/group. **b** Histogram showing the distribution of nuclear Hoechst sum intensity, with thresholds indicating separation between G1, S, and G2/M phases. **c** Quantification of the percentage of cells in each cell cycle phase. **d** Cell shape quantification (cellular width/cellular length) after Gal3 treatment. Data are shown as individual replicates with mean ±SEM (each experimental replicate corresponds to an independent culture performed from a different cell passage). In c, two-way ANOVA with uncorrected Fisher’s LSD multiple comparisons was performed. In d, one-way ANOVA with Dunnett’s multiple comparisons was performed and significant ANOVA differences are shown. P-values are expressed with 3 decimals.

**
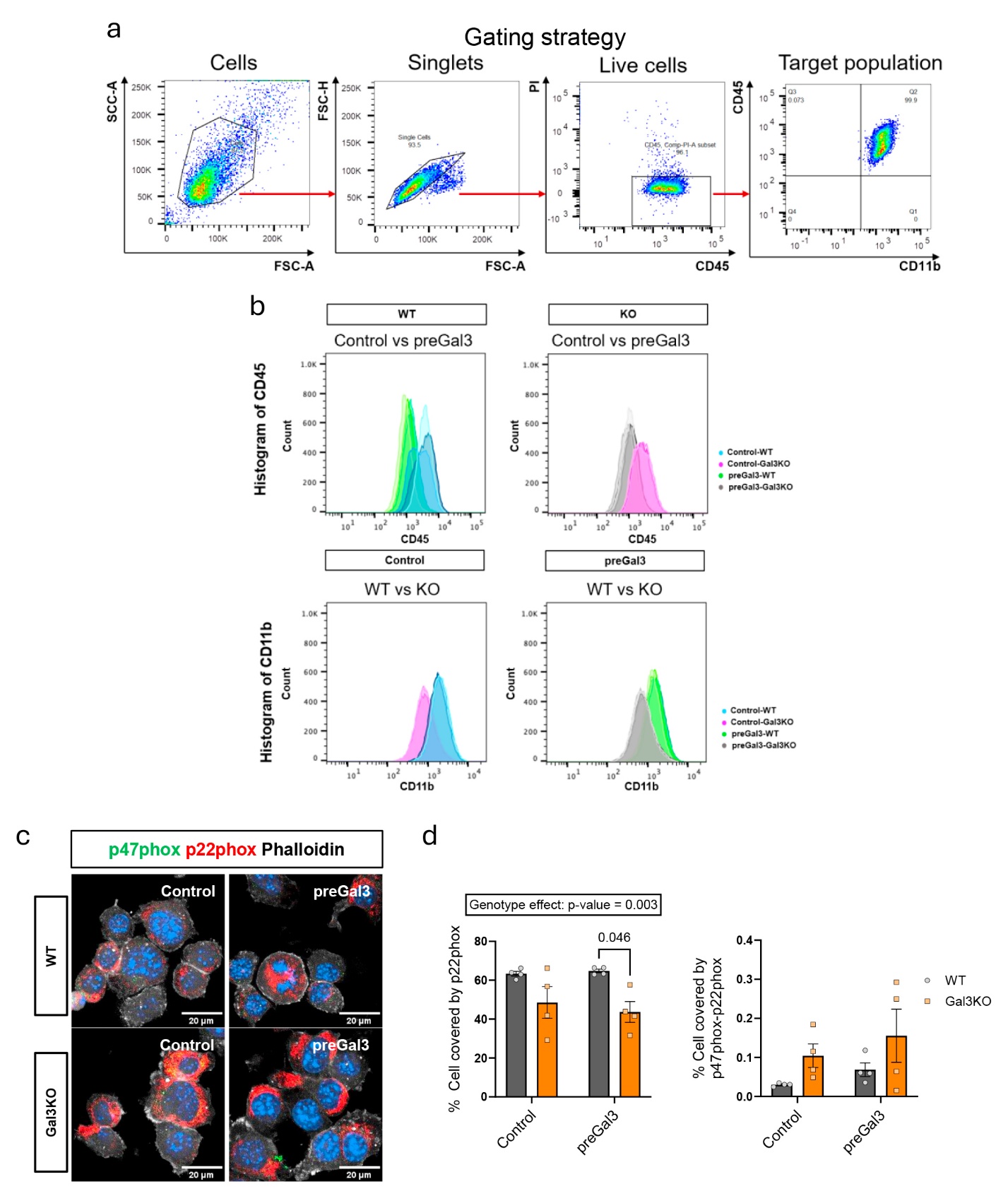
**

**Suppl. Fig. 3. Flow cytometry gating strategy with CD11b and CD45 histograms, and analysis of NOX2 subunits.**

**a** Gating strategy for flow cytometry analysis showing the sequential identification of singlets, live cells (propidium iodide-negative), and the final target population. **b** Histograms showing fluorescence intensity and cell counts for CD45 and CD11b, illustrating the separation of populations resulting from genotype- and treatment-dependent effects. **c** Representative immunofluorescence images of NOX2 subunits p22phox and p47phox. Scale bars, 20 µm. **d** Quantification of total p27phox content and p47phox-p22phox colocalization within BV2 cells. n=4 per group. Data are shown as individual replicates with mean ±SEM (each experimental replicate corresponds to an independent culture performed from a different cell passage). In d, two-way ANOVA with Tukey’s multiple comparisons was performed and significant ANOVA differences are shown. P-values are expressed with 3 decimals.

**
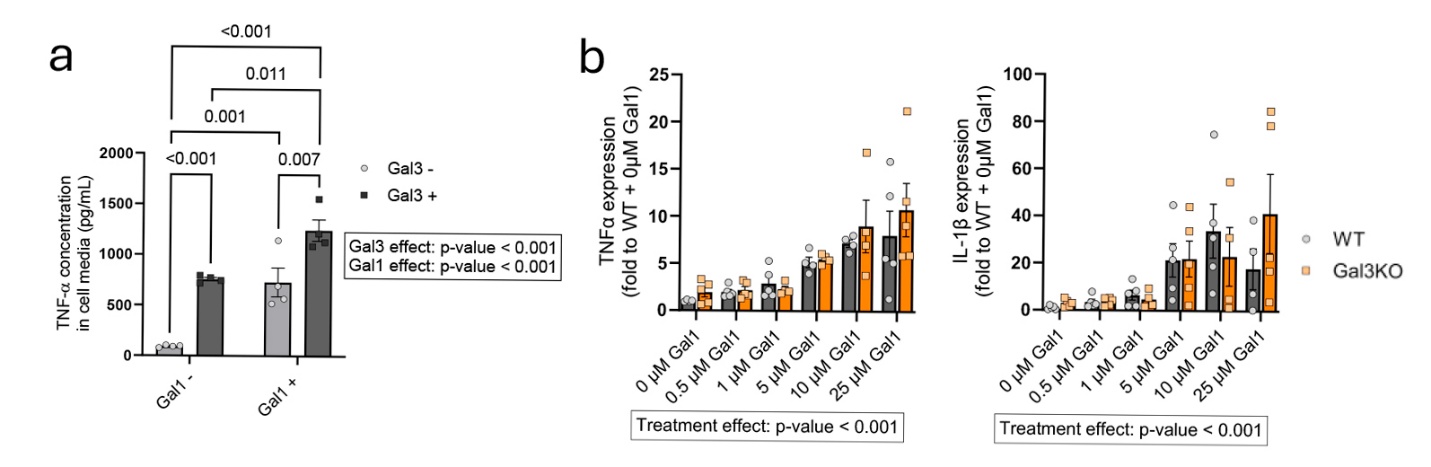
**

**Suppl. Fig. 4. TNFα after galectin-1 treatment in cell media and RT-qPCR of TNFα and IL-1β.**

**a** TNFα concentration in cell media of WT BV2 cells after Gal1 or/and Gal3 pretreatments. **b** RT-qPCR of IL-1β and TNFα of WT and Gal3KO BV2 cells after Gal1 pretreatment. Data are shown as individual replicates with mean ±SEM (each experimental replicate corresponds to an independent culture performed from a different cell passage). In a, two-way ANOVA with Tukey’s multiple comparisons was performed; in b, two-way ANOVA with Šidák’s multiple comparisons was performed; and significant ANOVA differences are shown. P-values are expressed with 3 decimals.
