# Supplementary material for: Galectin-3 shapes microglial phenotype through endogenous and exogenous mechanisms
Source: Front Cell Neurosci. 2025 Dec 18;19:1729776. doi: 10.3389/fncel.2025.1729776 (PMC12756361; doi:10.3389/fncel.2025.1729776)
Supplement: Supplementary file 3 [file Presentation_1.pptx]

## Slide 1
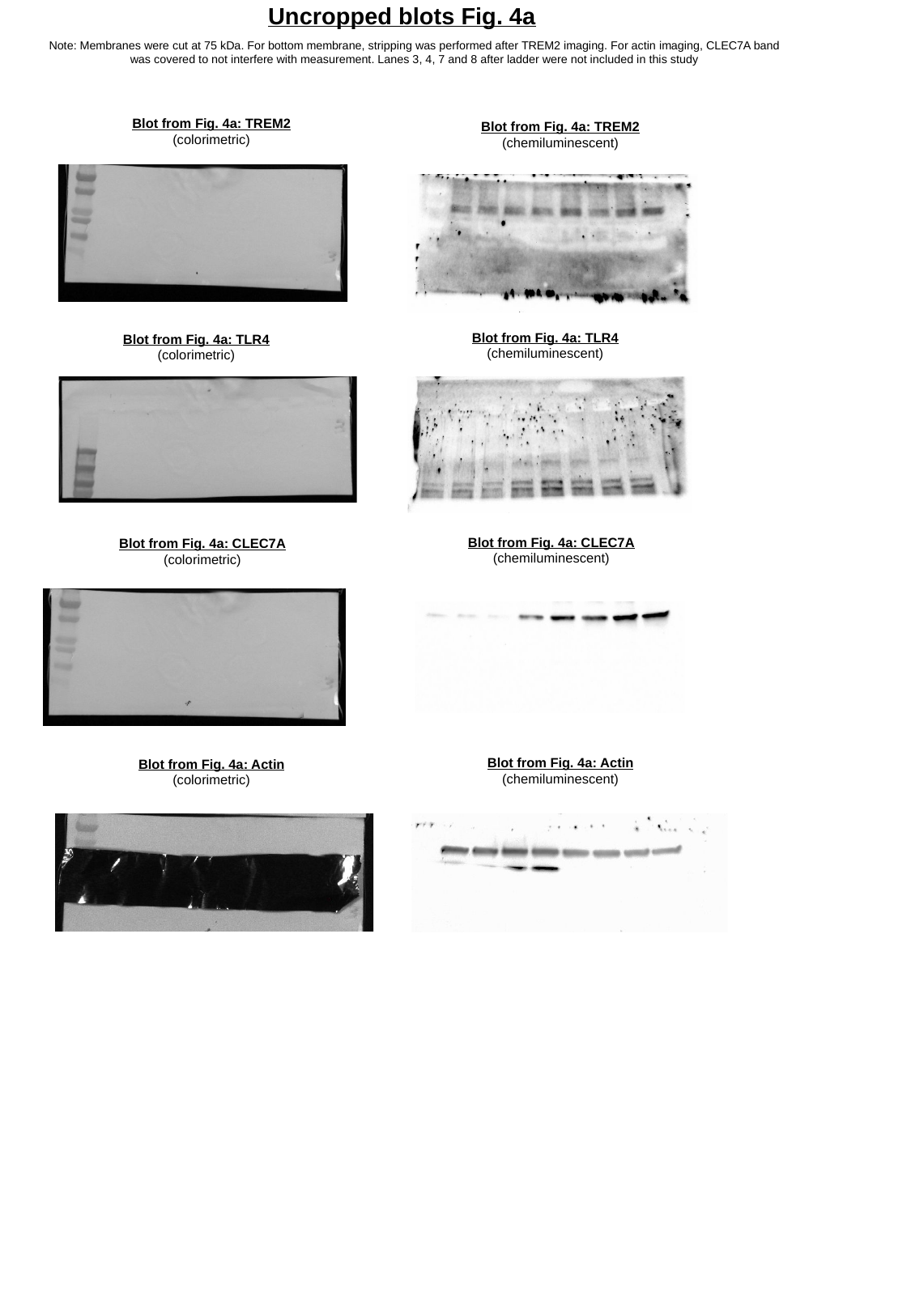

Uncropped blots Fig. 4a
Note: Membranes were cut at 75 kDa. For bottom membrane, stripping was performed after TREM2 imaging. For actin imaging, CLEC7A band was covered to not interfere with measurement. Lanes 3, 4, 7 and 8 after ladder were not included in this study
Blot from Fig. 4a: TREM2
(colorimetric)
Blot from Fig. 4a: TREM2
(chemiluminescent)
Blot from Fig. 4a: TLR4
(chemiluminescent)
Blot from Fig. 4a: TLR4
(colorimetric)
Blot from Fig. 4a: CLEC7A
(chemiluminescent)
Blot from Fig. 4a: CLEC7A
(colorimetric)
Blot from Fig. 4a: Actin
(chemiluminescent)
Blot from Fig. 4a: Actin
(colorimetric)

## Slide 2
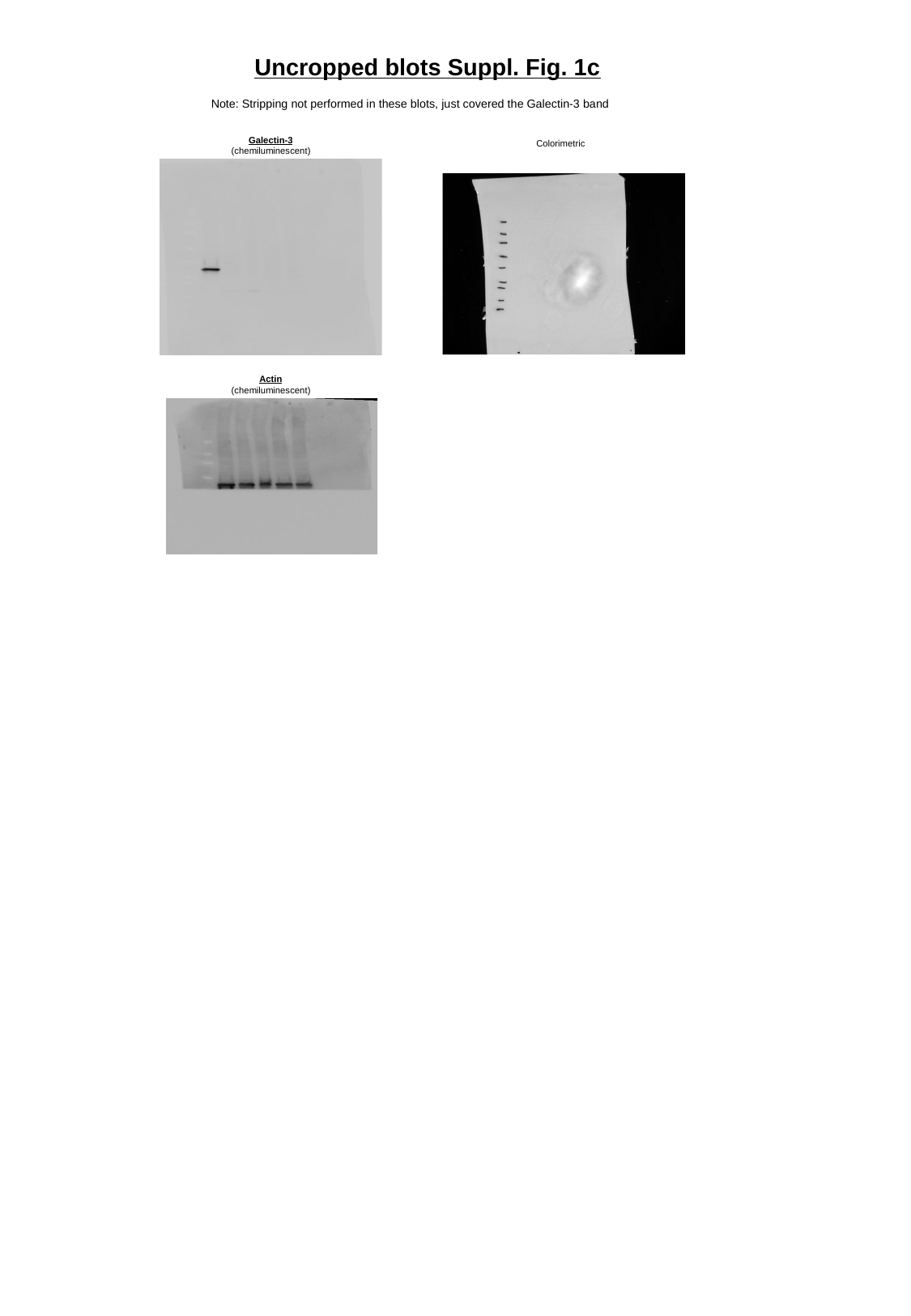

Uncropped blots Suppl. Fig. 1c
Note: Stripping not performed in these blots, just covered the Galectin-3 band
Galectin-3
(chemiluminescent)
Colorimetric
Actin
(chemiluminescent)
